# Genome Sequence of *Nitratireductor basaltis* Strain UMTGB225, a Marine Bacterium Isolated from a Green Barrel Tunicate in Bidong Island, Malaysia

**DOI:** 10.1128/genomeA.01015-14

**Published:** 2014-10-09

**Authors:** Muhd Danish-Daniel, Huan You Gan, Han Ming Gan, Nur Azna Saari, Gires Usup

**Affiliations:** a School of Fisheries and Aquaculture Sciences, Universiti Malaysia Terengganu, Kuala Terengganu, Terengganu, Malaysia; b Institute of Tropical Aquaculture, Universiti Malaysia Terengganu, Kuala Terengganu, Terengganu, Malaysia; c School of Science, Monash University Malaysia, Bandar Sunway, Petaling Jaya, Selangor, Malaysia; d School of Environmental and Natural Resource Sciences, Faculty of Science and Technology, Universiti Kebangsaan Malaysia, Bangi, Selangor, Malaysia

## Abstract

*Nitratireductor basaltis* strain UMTGB225 is a Gram-negative bacterium isolated from a marine tunicate found in Bidong Island, Terengganu, Malaysia. In this study, the genome of *Nitratireductor basaltis* UMTGB225 was sequenced to gain insight into the role of this bacterium and its association with tunicate hosts in a coral reef habitat.

## GENOME ANNOUNCEMENT

Several studies on bacterial communities associated with tunicates have been reported (1). However, the relationships between bacteria and host organisms are still not well understood. In order to better understand the tunicate-bacteria interactions, we sequenced the whole genome of *Nitratireductor basaltis* strain UMTGB225, which was isolated from a green barrel tunicate, *Didemnum molle*, collected from Bidong Island, Terengganu, Malaysia. The genus *Nitratireductor* belongs to the family *Phyllobacteriaceae* of the class *Alphaproteobacteria*. *Nitratireductor* strains were commonly reported to demonstrate the ability to convert nitrate to nitrite but are not able to reduce nitrite to nitrogen (2). To date*, Nitratireductor* has eight recognized species, and most of these were isolated from marine environments (2, 3), except for *Nitratireductor lucknowense*, which was isolated from pesticide-contaminated soil (4).

The genomic DNA of *N. basaltis* UMTGB225 was purified from an overnight Marine Broth 2216 (Difco) culture. The sequencing library was prepared using the TruSeq DNA sample preparation kit (Illumina, San Diego, CA). Sequencing of the *N. basaltis* UMTGB225 genome was performed using Illumina HiSeq 2000 (100-bp paired-end reads). Of the original sequencing data yield of 29,832,462 raw FASTQ paired-end reads, 2 million paired-end reads were randomly subsampled for downstream data processing. The subsampled reads were corrected for errors and *de novo* assembled into contigs with SPAdes Genome Assembler version 2.5.1 (5). Subsequently, the contigs were scaffolded and gap-filled using SSPACE version 2.0 (6) and GapFiller version 1.11 (7), respectively. The final assembly results in 5 contigs with an accumulated genome length of 3,551,806 bp and average coverage of 112×. Annotation for the genome was performed using the Prokka version 1.8 annotation pipeline, which comprised Aragorn version 1.2.36, Prodigal version 2.60, and RNAmmer version 1.2, leading to the prediction of 47 tRNAs, 3,451 open reading frames (ORFs), and 3 rRNAs (8–10). The predicted 16S rRNA from RNAmmer was queried with BLASTn (11) against the nucleotide collection database to validate its taxonomic assignment. Furthermore, InterProScan5 was used to provide additional annotation to the predicted protein sequences (12).

The predicted 16S rDNA sequence of strain UMTGB225 is 99.4% identical to that of *Nitratireductor basaltis* strain J3 (NCBI RefSeq accession number NR_044414), which was isolated from black beach sand from Soesoggak beach, Jeju Island, Korea (13). The genome is predicted to have genes involved in the reduction of nitrate to nitrite, potentially enabling strain UMTGB225 to utilize the nitrogenous compounds from the metabolic wastes produced by the tunicate host as an immediate source of energy. We observed genes involved in flagellum formation that may contribute to the mobility and host colonization of strain UMTGB225. The genome sequence of strain UMTGB225 and its curated annotation are important assets for better understanding the interaction of *Nitratireductor basaltis* with tunicates and other organisms in the marine environment and will open up new opportunities in the functional analysis of this species in the global biogeochemical nitrogen cycle.

### Nucleotide sequence accession number.

This whole-genome shotgun project has been deposited in DDBJ/EMBL/GenBank under the accession number JMQM00000000.
